# Nitrogen-Doped Biochar Derived from Starch for Enzyme-Free Colorimetric Detection of Uric Acid in Human Body Fluids

**DOI:** 10.3390/polym18010146

**Published:** 2026-01-05

**Authors:** Feihua Ye, Fan Chen, Yunhong Zhang, Yunwei Huang, Shasha Liu, Jiangfei Cao, Yanni Wu

**Affiliations:** 1 College of Environmental and Chemical Engineering, Zhaoqing University, Zhaoqing 526061, China; yefeihua@zqu.edu.cn (F.Y.); chenfan1730@gmail.com (F.C.); 19375824304@163.com (Y.Z.); huangyunwei202@163.com (Y.H.); liushasha@zqu.edu.cn (S.L.); 2 Guangdong Provincial Key Laboratory of Environmental Health and Land Resource, School of Environmental and Chemical Engineering, Zhaoqing University, Zhaoqing 526061, China; 3 New Energy and New Materials Research Center, Zhaoqing University, Zhaoqing 526061, China

**Keywords:** nitrogen-doped biochar, TMB, colorimetric assay, uric acid, biosensor

## Abstract

Uric acid (UA), a key end-product of human purine metabolism, serves as an important biomarker linked to multiple disorders. This study developed a novel enzyme-free colorimetric sensing platform based on starch-derived nitrogen-doped biochar (NC) for the highly sensitive and selective detection of UA in human body fluids. The NC material with a high specific surface area and abundant nitrogen active sites was prepared via a two-step strategy involving hydrothermal synthesis followed by high-temperature pyrolysis, using starch and urea as raw materials. Under mild conditions, the NC effectively catalyzes dissolved oxygen to produce reactive oxygen species (·O_2_^−^ and ^1^O_2_), which oxidize 3,3′,5,5′-tetramethylbenzidine (TMB) to a blue-colored oxidation product (TMBox). The presence of UA reduces TMBox to colorless TMB, leading to a measurable decrease in absorbance at 652 nm and enabling quantitative UA detection. Key reaction conditions were systematically optimized. Material characterization and mechanistic investigations confirmed the catalytic performance of the NC. The method demonstrated a wide linear response from 10 to 500 μmol·L^−1^, with a detection limit of 4.87 μmol·L^−1^, and demonstrated outstanding selectivity, stability, and reproducibility. Practical application in human serum and urine samples yielded results consistent with clinical reference ranges, and spike-recovery rates ranged from 95.5% to 103.6%, indicating great potential for real-sample analysis.

## 1. Introduction

Uric acid (UA), the terminal metabolite of human purine catabolism, is a crucial biomarker for the diagnosis and management of various diseases [1]. In healthy individuals, serum UA concentration typically ranges from 120 to 460 μM, while urinary UA levels are maintained between 1.4 and 4.4 mM [2,3]. Dysregulation of UA metabolism can lead to hyperuricemia, a direct cause of gout, kidney stones, and renal dysfunction [4]. Furthermore, numerous clinical studies have established a significant correlation between abnormal UA levels and chronic conditions such as cardiovascular disease, metabolic syndrome, and type 2 diabetes [5,6,7]. Consequently, developing rapid, accurate, cost-effective, and reliable methods for UA detection is essential for early disease screening and monitoring.

Currently, the primary methods for UA detection include enzymatic methods [8,9], chromatography [10,11], electrochemical methods [12,13], capillary electrophoresis [14], fluorescence spectroscopy [15,16], and chemiluminescence methods [17,18]. Among these, the enzymatic method is the most prevalent, relying on the specific catalysis by uricase. Quantification is achieved by measuring the hydrogen peroxide generated or the oxygen consumed during the reaction [9,19]. Despite its high selectivity, the application of enzymatic methods in resource-limited settings is constrained by the intrinsic limitations of biological enzymes, such as poor stability, high preparation and purification costs, stringent storage conditions (e.g., low temperature, protection from light), and sensitivity to environmental factors like pH and temperature [20]. Chromatographic techniques, exemplified by high-performance liquid chromatography, offer high accuracy and specificity but involve expensive instrumentation, require specialized operators and complex sample pretreatment, rendering them unsuitable for on-site rapid detection [21]. Electrochemical methods have garnered interest due to their high sensitivity, fast response, and potential for miniaturization. Nonetheless, they often suffer from electrode fouling or passivation by complex biological matrices, leading to poor reproducibility, and typically necessitate intricate electrode modification procedures [22]. Other methods, including capillary electrophoresis, fluorescence spectroscopy, and chemiluminescence, are often hampered by complicated sample preparation, sophisticated detection systems, or inadequate selectivity.

In recent years, enzyme-free colorimetric sensing technology has emerged as a promising approach in bioanalysis owing to its operational simplicity, low cost, and capacity for semi-quantitative visual readout [23,24,25]. These methods generally rely on catalytic materials to oxidize 3,3′,5,5′-tetramethylbenzidine (TMB), generating a color change. The reducing ability of the target analyte then decolorizes this product, enabling quantitative detection via UV-Vis spectrophotometry or smartphone-based imaging [25]. To date, various active materials have been developed for enzyme-free UA detection, such as noble metal nanoparticles (e.g., Au, Ag) [26,27], metal oxides (e.g., Co_3_O_4_, MnO_2_) [24,28], metal–organic frameworks (MOFs) [29], and polymetallic oxomolybdate [30]. Nevertheless, these materials present several limitations: noble metal nanoparticles are expensive and susceptible to aggregation [31]; certain metal-based materials may leach ions, raising concerns about biotoxicity and environmental impact [32]; and the synthesis procedures for many materials remain relatively complex.

Carbon-based materials, especially doped carbons, stand out among candidate materials due to their excellent chemical stability, high specific surface area, good biocompatibility, and tunable electronic structure [33,34]. Nitrogen doping, in particular, has proven to be an effective strategy for boosting the catalytic activity of carbon materials. The incorporated nitrogen atoms can modify the electron distribution of the carbon lattice, generate additional active sites, and improve interactions with substrate molecules [35,36]. Research indicates that graphitic nitrogen and nitrogen-containing functional groups in nitrogen-doped carbons act as effective active sites for catalytic oxidation processes [37,38]. This offers a distinct advantage for developing enzyme-free colorimetric methods for UA detection based on TMB oxidation catalysis. Nevertheless, most reported nitrogen-doped carbon materials are predominantly employed as peroxidase or oxidase mimics [39,40,41,42], with their application in enzyme-free colorimetric assays for UA detection in human body fluids remaining relatively unexplored.

**Figure 1:**
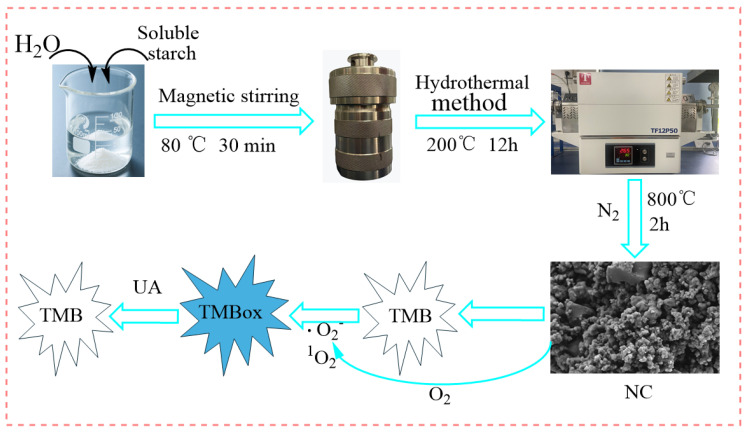
Preparation of NC and principle of NC-TMB enzyme-free colorimetric detection of UA.

This study developed a novel enzyme-free colorimetric sensing platform based on starch-derived nitrogen-doped biochar (NC) for the highly sensitive and selective detection of UA in human body fluids (Figure 1). Using starch and urea as green and low-cost precursors, NC with a porous structure, high specific surface area, and abundant nitrogen active sites was synthesized via a two-step strategy comprising hydrothermal treatment followed by high-temperature pyrolysis. The as-prepared NC efficiently catalyzes the generation of superoxide radical (O_2_^−^) and singlet oxygen (^1^O_2_) from dissolved oxygen under mild conditions. These reactive oxygen species oxidize TMB to form a blue-colored oxidation product (TMBox). UA reduces TMBox back to colorless TMB, resulting in a concentration-dependent decrease in absorbance at 652 nm, which serves as the signal for UA quantification. Key experimental parameters, including reaction temperature, TMB volume, NC dosage, reaction time, and solution pH, were systematically optimized. The NC material was comprehensively characterized using X-ray diffraction (XRD), scanning electron microscopy (SEM), surface area and porosity analysis (BET), and X-ray photoelectron spectroscopy (XPS). This work is expected to provide new insights for developing low-cost, high-performance enzyme-free biosensors and to advance their practical application in clinical diagnostics and health monitoring.

## 2. Materials and Methods

### 2.1. Reagents and Instruments

Reagents and chemicals used in this study included: soluble starch ((C_6_H_10_O_5_)_n_), urea (Urea), acetic acid (HAc), NaH_2_PO_4_·2H_2_O, Na_2_HPO_4_·12H_2_O, glucose (Glu), NaOH, hydrogen peroxide (H_2_O_2_), KCl, Na_2_CO_3_, and ascorbic acid (AA) from Guangzhou Chemical Reagent Factory (Guangzhou, China); uric acid (UA) from Shanghai McLean Biochemical Science & Technology Co., Ltd. (Shanghai, China); absolute ethanol from Xilong Chemical Co., Ltd. (Foshan, China); cysteine (Cys), p-benzoquinone (BQ), tert-butanol (TBA), glycine (Gly), aspartic acid (Asp), tyrosine (Tyr), dopamine (DA), 3,3′,5,5′-tetramethylbenzidine (TMB), and furfuryl alcohol (FFA) from Shanghai Aladdin Biochemical Technology Co., Ltd. (Shanghai, China); cholesterol (Chol) from Oddfoni Biotechnology Co., Ltd. (Nanjing, China); sodium acetate (NaAc) from West Asia Reagent; medium-chain triglycerides (C_39_H_74_O_6_, Trig) from Shanghai Yuanye Biotechnology Co., Ltd. (Shanghai, China); NH_4_Cl and Ca(CH_3_COO)_2_·H_2_O from Tianjin Komeo Chemical Reagent Co. (Tianjin, China). All reagents were of analytical grade, and deionized water was used throughout the experiments.

The UV–Vis absorption spectra were recorded on a Shimadzu UV-2600 spectrophotometer (Kyoto, Japan). The functional groups of the NC material were analyzed using a Shimadzu IRTracer-100 Fourier transform infrared (FT-IR) spectrometer (Kyoto, Japan). Its crystalline structure was examined by X-ray diffraction (XRD) on a Bruker D8 Advance diffractometer (Karlsruhe, Germany). Morphological observations were performed using a Carl Zeiss Supra 55 field-emission scanning electron microscope (SEM) (Oberkochen, Germany). The elemental composition and chemical states were determined by X-ray photoelectron spectroscopy (XPS) on a Thermo Scientific ESCALAB 250XI instrument (Waltham, MA, USA). The specific surface area, pore size distribution, and pore volume were obtained from N_2_ adsorption–desorption isotherms measured with a Quantachrome Autosorb-iQ analyzer (Boynton Beach, FL, USA). Sample preparation involved a 100 mL hydrothermal reactor (Xian Yichuang Co., Ltd. (Xi’an, China)) for the hydrothermal step and a TF12P50 vacuum/atmosphere tube furnace (Tianjin Tester Instrument Co., Ltd. (Tianjin, China)) for pyrolysis. Colorimetric reactions were carried out in a WE-2 thermostatic water-bath shaker (Tianjin Honour Instrumentation Co., Ltd. (Tianjin, China)).

### 2.2. Preparation of Starch-Based Nitrogen-Doped Biochar

Starch-based nitrogen-doped biochar (NC) was synthesized through a sequential hydrothermal-carbonization approach. Briefly, 5.0 g of soluble starch and 1.0 g of urea were dissolved in 50 mL of deionized water under magnetic stirring at 80 °C for 30 min. The homogeneous mixture was then transferred into a high-pressure hydrothermal reactor and subjected to hydrothermal treatment at 200 °C for 12 h [38]. After cooling, the resulting solid product was collected and alternately rinsed with anhydrous ethanol and deionized water until the supernatant reached neutral pH. The washed solid was subsequently dried in a vacuum oven at 80 °C for 5 h. Finally, the dried precursor was placed in a tube furnace and pyrolyzed at 800 °C for 2 h under a nitrogen atmosphere with a heating rate of 5 °C·min^−1^, followed by natural cooling to room temperature. The obtained black powder was denoted as NC and stored for further use. Starch-derived biochar (BC) was prepared following the aforementioned procedure without the addition of urea.

### 2.3. Colorimetric Determination of UA

Additionally, 35 μL of an 8 mmol/L TMB solution was thoroughly mixed with 2.5 mL of NaAc-HAc buffer (0.2 M, pH 4.6) in a centrifuge tube. Subsequently, 4 mg of NC was introduced, and the mixture was shaken in a water bath at 35 °C for 5 min. After reaction, the suspension was filtered through a 0.22 μm membrane. To the resulting filtrate, 200 μL of UA standard solutions at various concentrations were added, followed by shaking at 35 °C for 20 min. The UV-Vis spectra were then recorded over 400–800 nm, and the absorbance at 652 nm (A_652nm_) was measured. The absorbance difference ΔA was calculated as ΔA = A_0_ – A_n_, where A_0_ and A_n_ correspond to A_652nm_ in the absence and presence of UA, respectively. All the above experiments were repeated three times.

### 2.4. Pretreatment of Human Serum and Urine

Human serum and urine samples were obtained from three healthy volunteers. Serum samples were collected with assistance from Zhaoqing College Hospital and centrifuged at 4000 rpm for 15 min; the resulting supernatant was used directly for UA determination. Urine samples were diluted tenfold with deionized water prior to UA analysis.

## 3. Results and Discussion

### 3.1. Characterization of NC

**Figure 2:**
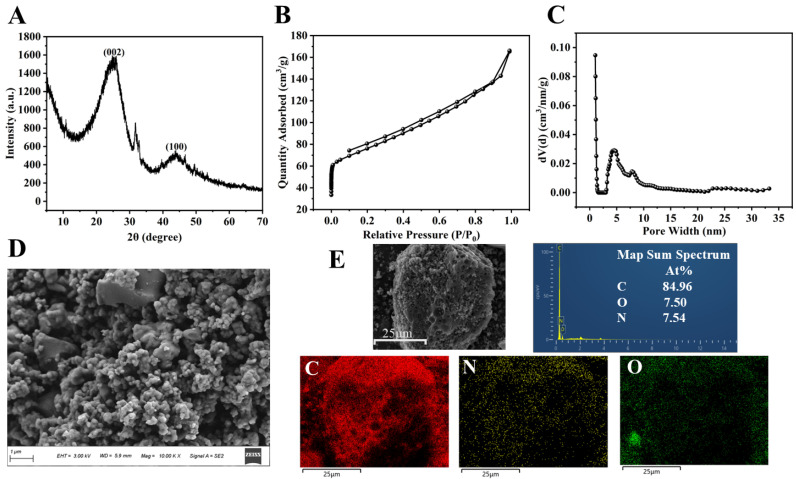
(**A**) XRD spectra of NC; (**B**) adsorption isotherm and (**C**) pore size distribution of NC; (**D**) SEM image of NC; (**E**) map sum spectrum and C, N, O, element distribution of NC.

**Figure 3:**
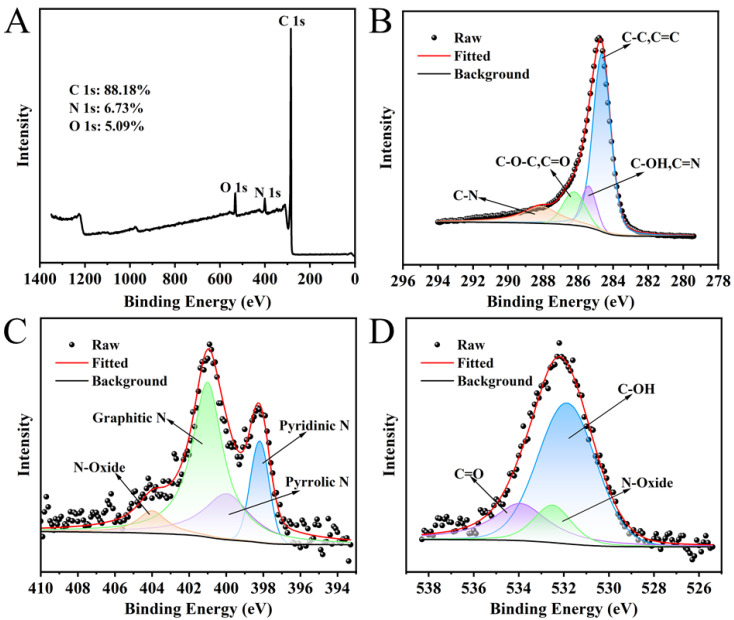
XPS (**A**), C 1 s (**B**), N 1 s (**C**) and O 1 s (**D**) spectra of NC.

The as-prepared NC was analyzed by a combination of techniques, including XRD, SEM, BET, and XPS; the corresponding data are depicted in Figure 2 and Figure 3. In the XRD pattern (Figure 2A), two broader diffraction peaks were observed at 25.82° and 43.78°, which were indexed to the (002) and (100) planes of graphitic carbon, thereby confirming its graphitic carbon structure [43]. The diffraction peak (26.5°) of the graphitic carbon (002) crystal plane was displaced to 25.82°, indicating that the N atom had doped into the carbon network [44]. The nitrogen adsorption isotherm of NC (Figure 2B) showed that NC had microporous and mesoporous structures. The Density Functional Theory (DFT) pore size distribution plots (Figure 2C) further show that the pore diameters are mainly distributed between 1.029 and 6.830 nm, with an average pore size of 1.030 nm. In addition, the specific surface area, micropore volume and total pore volume of NC were 272.861 m^2^·g^−1^, 0.049 cm^3^·g^−1^ and 0.258 cm^3^·g^−1^, respectively. The high specific surface area and hierarchical pore architecture (coexisting micropores and mesopores) contribute collectively to the enhanced catalytic performance: the large surface area offers abundant nitrogen-active sites for reactant adsorption, while the suitable pore size distribution facilitates efficient diffusion and interaction between TMB and O_2_ molecules, thereby promoting TMB oxidation. The SEM image (Figure 2D) reveals that the NC is composed of irregularly shaped particles that aggregate to form a porous morphology. The surface of the particles appears relatively smooth. The elemental distribution was analyzed using mapping (Figure 2E). The results indicate that carbon, nitrogen, and oxygen are uniformly dispersed. Specifically, the homogeneous spatial distribution and consistent signal intensity of nitrogen confirm its successful incorporation into the carbon matrix. The XPS spectrum (Figure 3A) of NC displays three distinct peaks at approximately 285, 401, and 533 eV, corresponding to C 1s, N 1s, and O 1s, respectively. The C, N and O elemental contents of NC were 88.18%, 6.73% and 5.09%, respectively. The peak-fitting analysis of the C 1s spectrum is presented in Figure 3B. The spectrum is deconvoluted into four component peaks located at binding energies of 284.65 eV (assigned to C-C/C=C), 285.40 eV (C-OH/C=N), 286.23 eV (C-O-C/C=O), and 288.12 eV (C-N) [44]. The N 1s spectrum (Figure 3C) was fitted with four peaks assigned to pyridinic N (398.20 eV), pyrrolic N (399.97 eV), graphitic N (401.01 eV), and N-oxide (404.00 eV) [45,46]. The O 1s spectrum (Figure 3D) exhibits peaks at 531.86 eV (C–OH), 532.50 eV (N–oxide), and 533.94 eV (C=O). XPS analyses showed that N was successfully incorporated into the carbon framework, which is consistent with the XRD results.

### 3.2. Feasibility of UA Colorimetric Assay

Five centrifuge tubes were prepared and labeled a-e. 2.5 mL NaAc-HAc buffer solution (NHBS, 0.2 M, pH = 4.0) was added each centrifuge tube, and then the reagents added to it were as follows:

- Centrifuge tube a: 200 μL H_2_O_2_ (200 μmol·L^−1^).
- Centrifuge tube b: 200 μL H_2_O_2_ (200 μmol·L^−1^), 60 μL TMB (8 mmol·L^−1^).
- Centrifuge tube c: 60 μL TMB (8 mmol·L^−1^), 8 mg NC.
- Centrifuge tube d: 200 μL H_2_O_2_ (200 μmol·L^−1^), 60 μL TMB (8 mmol·L^−1^), 8 mg NC.
- Centrifuge tube e: 60 μL TMB (8 mmol·L^−1^), 8 mg NC.

**Figure 4:**
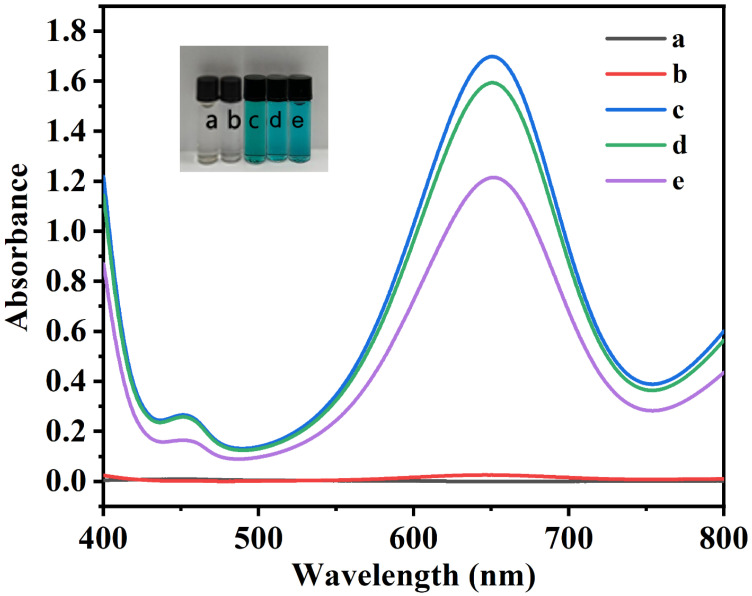
UV-Vis spectra of (a) H_2_O_2_, (b) H_2_O_2_ + TMB, (c) TMB + NC, (d) H_2_O_2_ + TMB + NC, (e) TMB + NC + UA. The inset shows photographs of the corresponding solutions under sunlight. Experimental conditions: 2.5 mL 0.2 M NHBS (pH = 4.0), 200 μL 200 μmol·L^−1^ H_2_O_2_, 60 μL 8 mmol·L^−1^ TMB, 8 mg NC, 200 μL 1 mmol·L^−1^ UA.

After thorough vortexing, all reaction tubes were incubated at 30 °C in a water bath for 5 min. The mixtures were subsequently filtered through a 0.22 μm membrane. To the filtrate in tube (e), 200 μL of a 1 mmol·L^−1^ UA solution was added, followed by a second incubation under identical conditions (30 °C) for 10 min. Finally, the UV-Vis absorption spectra of the resulting solutions were recorded across the 400–800 nm range (Figure 4), with the following observations:

- No absorption at 652 nm was observed in the H_2_O_2_ system (Figure 4(a)).
- The colorless solution and a minimal A_652nm_ were observed in the H_2_O_2_ + TMB system (Figure 4(b)), confirming the limited oxidizing capacity of H_2_O_2_ toward TMB.
- The color of the solutions in both the TMB + NC (Figure 4(c)) and H_2_O_2_ + TMB + NC (Figure 4(d)) systems was blue, and the former has a slightly higher A_652nm_ than the latter. This result demonstrates the ability of the TMB + NC system to achieve satisfactory color development in the absence of H_2_O_2_.
- The addition of UA to the filtrate of the TMB + NC system resulted in a significant decrease in A_652nm_ (Figure 4(e)). This result indicates that UA can reduce the blue TMBox back to TMB, thereby confirming the feasibility of the colorimetric UA detection method based on the reduction in TMBox as monitored at 652 nm.

### 3.3. Optimization of Color Development Conditions and Stability

**Figure 5:**
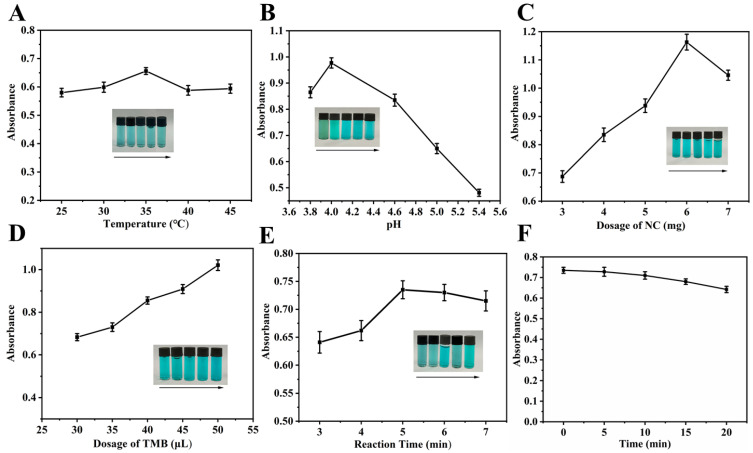
The color development effect of (**A**) reaction temperature (2.5 mL 0.2 M NHBS (pH = 5.0), 4 mg NC, 40 μL 8 mmol·L^−1^ TMB, 5 min), (**B**) reaction environment pH (4 mg NC, 40 μL 8 mmol·L^−1^ TMB, 35 °C, 5 min, 2.5 mL 0.2 M NHBS (pH = 3.8, 4.0, 4.6, 5.0, 5.4)), (**C**) NC dosage (2.5 mL 0.2 M NHBS (pH = 4.6), 40 μL 8 mmol·L^−1^ TMB, 35 °C, 5 min), (**D**) TMB dosage (2.5 mL 0.2 M NHBS (pH = 4.6), 4 mg NC, 35 °C, 5 min) and (**E**) reaction time (2.5 mL 0.2 M NHBS (pH = 4.6), 4 mg NC, 35 μL 8 mmol·L^−1^ TMB, 35 °C), (**F**) NC-TMB color development stability (2.5 mL 0.2 M NHBS (pH = 4.6), 4 mg NC, 35 μL 8 mmol·L^−1^ TMB, 35 °C, 5 min). Inset: the photos of corresponding solutions in sunlight.

A systematic optimization of key parameters (temperature, pH, NC dosage, TMB dosage, and reaction time) was conducted to achieve optimal system color development, with the results detailed in Figure 5. As shown in Figure 5A, the A_652nm_ of the system was affected by the reaction temperature, with the maximum value at 35 °C in the range of 25–45 °C. This was because the catalytic activity of NC was inherently slightly low at temperatures below 35 °C, while at temperatures above 35 °C, its efficiency was impaired by a reduction in dissolved oxygen. Hence, the optimal reaction temperature was determined to be 35 °C. As confirmed in Figure 5B, the pH of the system (within the range of 3.0 to 5.4) significantly influenced the catalytic activity of NC. When the pH < 4, the excessive catalytic activity of NC led to over-oxidation of TMB, causing the solution color to shift from blue-green to green and a consequent decrease in A_652nm_. Conversely, when the pH > 4, the A_652nm_ decreased significantly with increasing pH. As a result, the optimal pH was determined to be 4.6. The effect of NC dosage on the system is shown in Figure 5C. The A_652nm_ of the system initially increased and then decreased with the increasing amount of NC. Since absorbance values exceeding 1 were found to compromise the linear relationship between UA concentration and A_652nm_, 4 mg of NC was selected as the optimal dosage. As seen in Figure 5D, the A_652nm_ of the system exhibited a dose-dependent increase with TMB volume over the range of 30–50 μL. A volume of 35 μL was selected to prevent the absorbance from becoming excessive, which could adversely affect the detection limit for UA. The effect of reaction time on the A_652nm_ of the system is depicted in Figure 5E. The A_652nm_ initially increased and then slightly decreased as the reaction time increased from 3 to 7 min. The observed decrease in A_652nm_ was due to over-oxidation of the TMB when reaction time exceeded 5 min. Therefore, a reaction time of 5 min was chosen as optimal.

The stability of the NC–TMB chromogenic system was confirmed under optimized conditions, with the A_652nm_ showing a relative standard deviation (RSD) of less than 1.78% over 10 min (Figure 5F). This indicates that the reaction medium maintained good stability after the removal of NC by filtration, thus providing sufficient time for subsequent measurements without the addition of termination or stabilizing agents.

### 3.4. Optimization of UA Reduction Time

**Figure 6:**
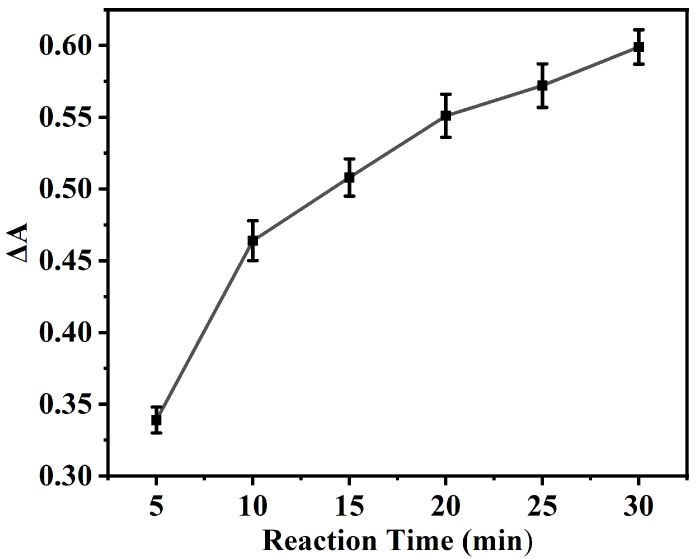
The influence of the UA reduction duration on ΔA. Experimental conditions: 2.5 mL 0.2 M NHBS (pH = 4.6), 35 μL 8 mmol·L^−1^ TMB, 4 mg NC, 35 °C, 5 min, 200 μL 400 μmol·L^−1^ UA.

The influence of the UA reduction duration on ΔA is presented in Figure 6, wherein the ΔA value undergoes a rapid initial ascent within the first 10 min, subsequently approaching a plateau. A 20 min reduction time was ultimately adopted, equilibrating the considerations of analytical efficiency and signal stability. This allows for the capture of a substantial signal alteration while circumventing an undue prolongation of the reaction time.

### 3.5. Stability of NC

**Figure 7:**
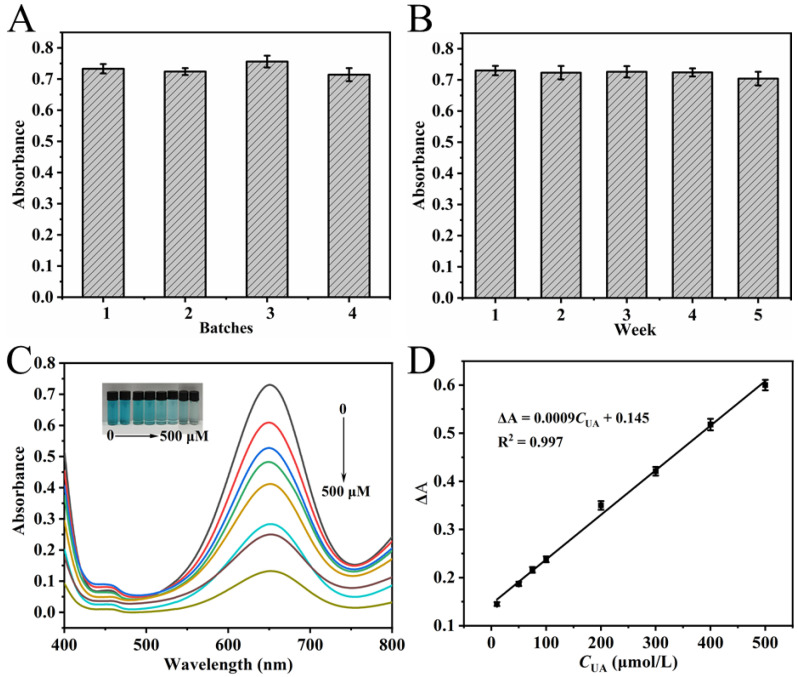
Stability evaluation of NC under different batches (**A**) and storage durations (**B**). Experimental conditions: 2.5 mL 0.2 M NHBS (pH = 4.6), 35 μL 8 mmol·L^−1^ TMB, 4 mg NC, 35 °C, 5 min. (**C**) UV–Vis spectra of oxidized TMB solutions in response to varying UA concentrations; (**D**) Corresponding linear calibration curve for UA detection. Experimental conditions for (**C**,**D**): 2.5 mL 0.2 M NHBS (pH = 4.6), 35 μL 8 mmol·L^−1^ TMB, 4 mg NC, 35 °C, 5 min, UA 10–500 μmol·L^−1^, 20 min.

A comprehensive evaluation of NC stability was performed by assessing the colorimetric results from different batches and from a single batch following different storage periods, all under optimal color development conditions. In Figure 7A, minimal variation was observed in the absorbance at 652 nm (RSD = 2.5%) among the color development systems of four NC batches prepared concurrently. This result indicates that the activity of different batches of NC remains consistent. Moreover, when a single batch of NC was stored for 1 to 5 weeks, the absorbance of its color development system at 652 nm showed minimal change, with an RSD of 1.4% (Figure 7B). This indicates that the activity of the NC remains stable after long-term storage. The excellent stability of the synthesized NC, as evidenced by these results, supports its potential for reliable, long-term use in colorimetric detection.

### 3.6. UA Standard Calibration Curve and Detection Limit

Under optimal chromogenic conditions, 200 μL of UA solutions with varying concentrations (10–500 μmol·L^−1^) were added to the chromogenic system and subjected to hydrothermal treatment at 35 °C for 20 min. UV–Vis spectra of the resulting solutions were subsequently recorded between 400 and 800 nm (Figure 7C). As illustrated in the inset of Figure 7C, the A_652nm_ decreased gradually with increasing UA concentration, consistent with visible fading of the solution color. A linear correlation was established between the change in absorbance (ΔA) and UA concentration over the range of 10–500 μmol·L^−1^ (Figure 7D). The calibration curve was fitted with the equation: ΔA = 0.0009*C*_UA_ + 0.145 (R^2^ = 0.997). The method demonstrated high sensitivity, with a limit of detection (LOD) determined to be 4.87 μmol·L^−1^, calculated based on 3σ/s (where s represents the calibration curve slope and σ denotes the standard deviation from seven replicate measurements).

Method precision was evaluated using UA solutions at three concentrations (15 μmol·L^−1^, 50 μmol·L^−1^, and 200 μmol·L^−1^) within the linear range. Each concentration was subjected to five successive measurements and the RSDs were determined to be 2.9%, 2.5%, and 1.7%, confirming the high precision of the method.

As summarized in Table 1, the NC enzyme-free colorimetric method demonstrates analytical performance comparable to existing UA detection techniques across key parameters including linear range, detection limit, and analysis time. Notably, this method operates without requiring metals, enzymes (including natural enzymes and nanozymes), or H_2_O_2_. Moreover, the detection procedure is faster than enzyme-based assays, and the preparation of NC is straightforward and environmentally benign. Consequently, the NC enzyme-free colorimetric strategy demonstrates rapid response, low cost, high sensitivity and excellent eco-compatibility. These advantages collectively render it a promising analytical platform for UA determination.

### 3.7. Selectivity of UA Sensing

The selectivity of the NC enzyme-free colorimetric method for UA detection was evaluated by testing potential interfering substances commonly found in serum and urine, including amino acids, biomolecules, and various ions.

**Figure 8:**
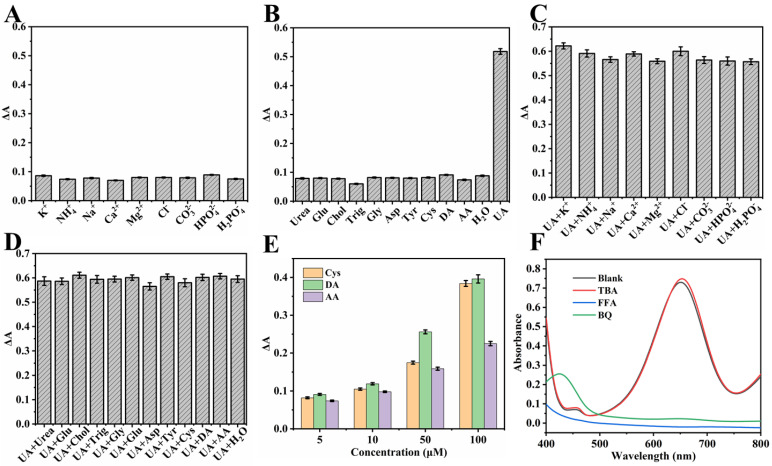
(**A**–**D**) Selectivity of NC enzyme-free colorimetric method (the concentrations used were 400 μmol·L^−1^ for UA, 5 μmol·L^−1^ for DA, AA, and Cys, and 500 μmol·L^−1^ for all other interfering substances). (**E**) Effect of varying concentrations of DA, AA, and Cys on the system absorbance. (**F**) Influence of UA, BQ, TBA, and FFA on the color development of the NC-TMB system. Experimental conditions: 2.5 mL of 0.2 M NHBS (pH = 4.6), 4 mg BQ or 200 μL FFA or 200 μL TBA, 35 μL 8 mmol·L^−1^ TMB, 4 mg NC, 35 °C, 5 min.

As shown in Figure 8A,B, the ΔA of the system was measured after the addition of 500 μmol·L^−1^ K^+^, Na^+^, Ca^2+^, Mg^2+^, NH_4_^+^, Cl^−^, CO_3_^2−^, HPO_4_^−^, H_2_PO_4_^−^, Gly, Asp, Tyr, Trig, Urea, Chol, Glu, 5 μmol·L^−1^ DA, AA, Cys, and 400 μmol·L^−1^ UA. The results indicated that only UA caused a significant decrease in the absorbance of the system at the aforementioned concentrations. Furthermore, when the above substances were added simultaneously with 400 μmol·L^−1^ UA, no interference was observed in the system’s ΔA, as shown in Figure 8C,D. Further studies revealed that AA, DA, and Cys significantly affected the detection system only when their concentrations exceeded 10 μmol·L^−1^ (Figure 8E). Fortunately, given their considerably lower concentrations in serum and urine compared to UA [25], AA, DA, and Cys did not interfere with the detection, thus demonstrating the good selectivity of the developed assay.

### 3.8. UA Detection Mechanism

**Figure 9:**
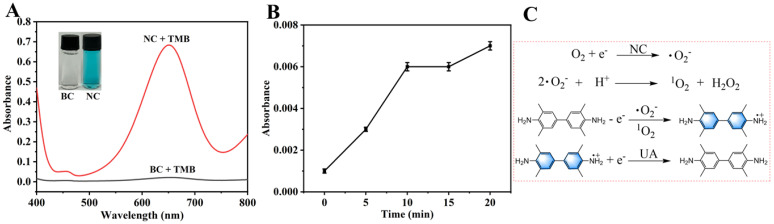
(**A**) Comparison of the catalytic performance between BC and NC; (**B**) the effect of air exposure duration on the oxidation of TMB; (**C**) free radicals and color development reaction equations.

To investigate the mechanism of the NC-TMB chromogenic system, free radical scavengers including TBA, BQ, and FFA were employed to quench ·OH, ·O_2_^−^, and ^1^O_2_, respectively. Under optimal chromogenic conditions, 4 mg of BQ, or 200 μL of FFA or TBA, was added to the system before the introduction of TMB. The UV-Vis spectra (Figure 8F) revealed that the A_652nm_ remained nearly unchanged after the addition of TBA. In contrast, a significant reduction in absorbance at 652 nm—reaching nearly zero—was observed upon the introduction of BQ and FFA. As shown in Figure 9A, under identical conditions, only NC was capable of catalytically oxidizing TMB to TMBox, accompanied by a distinct color change in the solution from colorless to blue. This indicates that nitrogen doping is essential for the observed catalytic activity. In Figure 9B, TMB remained unoxidized after 20 min of air exposure in the absence of NC, with no significant change in absorbance at 652 nm. This demonstrates that air alone is insufficient to drive the chromogenic reaction of TMB. These results indicate that ·O_2_^−^ and ^1^O_2_ play a predominant role in promoting the chromogenic process. This phenomenon can be attributed to the active sites in NC, such as graphitic nitrogen and nitrogen-containing functional groups [37,38], which facilitate electron transfer to dissolved oxygen, generating ·O_2_^−^. The resulting ·O_2_^−^ subsequently reacts with H^+^ to yield ^1^O_2_ [51]. The operational mechanism for UA detection is depicted in Figure 1, which begins with NC catalyzing dissolved oxygen to produce ·O_2_^−^ and ^1^O_2_. These species then oxidize the colorless substrate TMB to form a blue TMBox product. In the key detection step, UA acts as a reducing agent, reverting blue TMBox to its colorless state (the reaction equation is shown in Figure 9C). This cascade culminates in a measurable drop in A_652nm_, which directly correlates with and enables the quantification of UA concentration.

### 3.9. Actual Urine Sample Testing

The novel NC enzyme-free colorimetric assay was evaluated via three parallel determinations of serum and urine samples from three healthy adult volunteers and spiked recovery experiments. As summarized in Table 2, the measured serum UA concentrations (380.70, 257.94, and 307.46 μmol·L^−1^) and urinary UA levels (3.22, 1.70, and 2.42 mmol·L^−1^) all fell within their established clinical reference ranges (120–460 μmol·L^−1^ and 1.4–4.4 m mol·L^−1^, respectively). The method validation results showed that the spiked recoveries for all samples ranged from 95.5% to 103.6%, with an RSD of less than 3%. These results indicate that the method is accurate, reliable, and highly precise for determining UA in serum and urine. For practical applications such as home-testing devices, monitoring urinary UA presents a more feasible alternative, as serum testing involves a more complex sampling procedure.

## 4. Conclusions

In summary, we successfully constructed an enzyme-free colorimetric sensing platform based on starch-derived nitrogen-doped biochar for the rapid, sensitive, and selective detection of uric acid. The NC material was synthesized via a green and low-cost method, exhibiting excellent catalytic activity and stability. Under optimal conditions, the assay displayed a wide linear range, a low detection limit, and strong anti-interference capability. Mechanistic studies revealed that NC catalyzes dissolved oxygen to generate ·O_2_^−^ and ^1^O_2_, which drive the chromogenic reaction of TMB. UA induces a signal switch by reducing blue TMBox to colorless TMB. Accurate and reliable results from real serum and urine sample analyses validate the method’s promising application potential in clinical diagnosis and health monitoring.

## Figures and Tables

**Table 1:** Comparison of the NC enzyme-free colorimetric assay and other methods for UA analysis.

Materials	Enzyme	Linear Range (μmol·L^−1^)	LOD (μmol·L^−1^)	Detection Time (min)	Reference
Fe/Mo DSACs	uricase	0.5–200	0.13	45	[8]
TMB-CoP/NF	peroxidase mimic	1–200	1.00	40	[19]
CaF_2_/MnO_2_	uricase	0.3–70	0.137	55	[25]
CoMnO_3_	uricase	0.6–200	0.38	25	[47]
BSA@Au nanoclusters	uricase	0.5–50	0.39	75	[48]
CaF_2_/MnO_2_	no enzyme	0.1~30	0.039	3	[25]
AuNRs-MnO_2_-KI	no enzyme	0.8~30	0.76	15	[26]
		30~300	2.04		
Ag/Au nanorods	no enzyme	0.1~1.0	0.065	2	[27]
MnO_2_ nanosheets	no enzyme	0.5~30	0.21	20	[28]
Cu(II)-complex	no enzyme	50–500	4.6	6	[49]
Holey MoS_2_	no enzyme	400–7000	5.62	0.8	[50]
NC	no enzyme	10–500	4.87	25	This work

**Table 2:** Analysis and spike recovery of actual serum and urine samples.

Samples	Measurement Results (μmol·L^−1^)	Measured Quantity Average (μmol·L^−1^)	Marked Quantity (μmol·L^−1^)	Total Amount Measured (μmol·L^−1^)	Recovery Rate (%)	RSD (%)
Serum 1	380.25	380.70	100	476.20	95.5	1.20
	376.36					
	385.49					
Serum 2	260.28	257.94	100	360.13	102.2	2.23
	251.37					
	262.16					
Serum 3	302.60	307.46	100	406.31	98.9	1.43
	308.58					
	303.20					
Urine 1	316.54	322.02	100	425.65	103.6	1.67
	322.22					
	327.30					
Urine 2	164.44	169.64	100	267.78	98.14	2.95
	174.44					
	170.03					
Urine 3	246.60	242.46	100	340.25	97.8	1.66
	238.56					
	242.22					

## Data Availability

The data presented in this study are available on request from the corresponding author due to privacy.
